# Acute thromboembolism in a solitary kidney: revascularization by means of isolated transcatheter thrombus aspiration

**DOI:** 10.1259/bjrcr.20150366

**Published:** 2016-05-17

**Authors:** Nelson Neto, Joaquin Gil Romero, Juan Manuel Sanchis, Jorge Guijarro, Julio Palmero da Cruz

**Affiliations:** ^1^ Radiology Department, Centro Hospitalar de Lisboa Ocidental, Lisbon, Portugal; ^2^ Radiology Department, Hospital Clínico Universitario de Valencia, Valence, Spain

## Abstract

Acute renal infarction occurs usually secondary to thromboembolism rather than *in situ* thrombosis, with atrial fibrillation being the main predisposing factor. Its non-specific presentation, being similar to renal colic and pyelonephritis, often leads to diagnostic and treatment delays. Prompt diagnosis and treatment are crucial for the outcome, as the precise therapeutic window remains unclear. Renal Doppler ultrasound is the optimal initial diagnostic study, which, if inconclusive, should be followed by contrast-enhanced CT. Despite the lack of specific guidelines, treatment is mainly based on anticoagulation and percutaneous endovascular revascularization therapy; the latter includes pharmacomechanical thrombectomy; intra-arterial thrombolysis alone or in combination with thrombus aspiration; and angioplasty, with or without stenting. We present a case of renal thromboembolism diagnosed early during the postoperative period in a patient with a single functioning kidney. Renal arterial flow restoration was achieved by means of transcatheter thrombus aspiration. This is the first report showing the effectiveness of this procedure alone as an alternative to those used so far.

## Clinical presentation

A 75-year-old male, with a history of right nephrectomy 3 years earlier owing to clear cell carcinoma and resection of multiple metastases (inferior pole of the contralateral kidney, ipsilateral adrenal gland and mesentery) 1 year earlier, was admitted electively for left lung lobectomy owing to metastases. Moreover, the patient had a history of prostate carcinoma, which remained stable after radiation and hormone therapy; long-term arterial hypertension; dyslipidemia; diabetes mellitus; chronic atrial fibrillation and Stage 4 chronic kidney disease with a baseline estimated glomerular filtration rate of 25.4 ml/min/1.73 m^2^. Approximately 48 h after surgery, the patient presented with anuria, deterioration of renal function with a decrease in the estimated glomerular filtration rate to 11.6 ml/min/1.73 m^2^ and elevation of serum lactate dehydrogenase to 2096 U/l (reference range 240–480 U/l).

## Investigations/imaging findings

**Figure 1.**
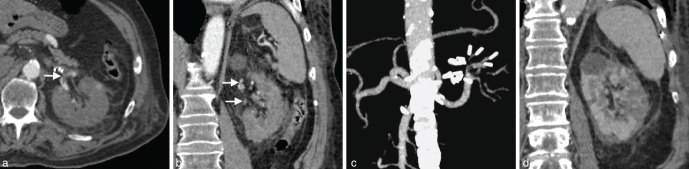
(a) Arterial-phase contrast-enhanced CT axial image shows a filling defect in the distal main trunk of the left renal artery corresponding to thromboembolic material (arrow). (b) Extension to both polar superior and inferior renal artery branches is also seen in the coronal view (arrows), the latter being completely occluded. (c) Three-dimensional maximum intensity projection reconstructed image better obviates the features previously described. (d) Large wedge-shaped areas of non-enhancing renal parenchyma are clearly depicted in the nephrographic phase of the same contrast-enhanced CT scan, consistent with extensive infarction.

**Figure 2.**
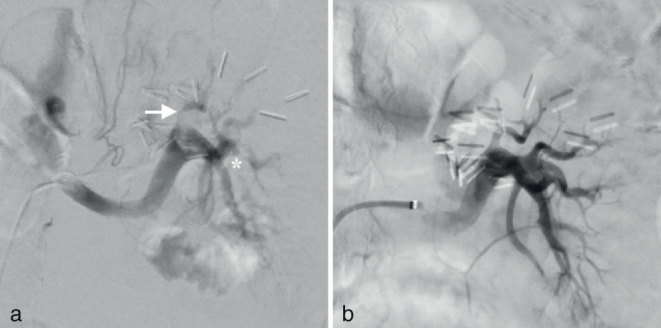
(a) Selective arteriography of the left kidney confirmed the subtotal obstruction of a polar superior branch of the renal artery (arrow) as well as an abrupt interruption of flow in a polar inferior branch (asterisk) corresponding to occlusion by an embolus. (b) After selective transcatheter thrombus aspiration, complete restoration of renal arterial flow is demonstrated in the final angiogram.

An ultrasound scan was negative for obstructive nephrolithiasis and raised a suspicion for renal infarction. Presumably owing to both technical limitations of the ultrasound device available in the emergency room and the decreased perfusion of the kidney associated with low blood flow velocity within the renal arteries, the Doppler evaluation was inconclusive. Therefore, a contrast-enhanced CT (CECT) scan for diagnostic confirmation seemed justifiable, despite the well-known additional damage to the kidney secondary to the use of i.v. contrast medium. For that purpose, only 80 cc of non-ionic iodinated contrast medium followed by 40 cc of saline solution was administered intravenously before volumetric scanning in both arterial and nephrographic phases. A filling defect was seen in the distal main trunk of the left renal artery that extended into the proximal segment of two of its branches (Figure 1a–c) as well as multiple non-enhancing areas involving more than 50% of the remaining renal parenchyma (Figure 1d), all consistent with extensive non-perfused parenchymal zones due to acute thromboembolism. Renal angiogram performed 12 h later by means of selective catheterization of the renal artery with a 4-Fr Cobra 2-shaped catheter (Terumo, Tokyo, Japan) introduced through a 6-Fr guiding sheath (Cook, Bloomington, IN) over a 0.035-inch hydrophilic guidewire (Terumo, Japan) with angled tip by right femoral approach confirmed a subtotal occlusion of the superior branch of the renal artery and a more distal inferior branch by thromboembolic material (Figure 2a).

## Treatment

Several reasons led to a treatment delay of 12 h. First, the definite diagnosis was achieved during the night, when only one urologist was available to consider the risks and benefits of each possible treatment option for a fairly complex clinical situation. Owing to the risk of bleeding in the postoperative period, fibrinolytic therapy was avoided. After discussion with the interventional radiology team, endovascular rescue of the kidney seemed to be the best therapeutic approach aiming to restore renal function in this patient with a single functioning kidney, previously allowing him to live free of dialysis. The fact that only segmental branches were occluded made the interventional radiologists expect partial recovery of renal function on the basis of some published data.^1^ In addition, expecting the thrombus to still be quite fresh, they considered transcatheter aspiration alone to likely restore renal perfusion. However, the team had never performed this technique in such context before and some research was required. Moreover, the coagulation status of the patient needed to be corrected and close family members had to be consulted in order to obtain a signed informed consent form before performing the procedure described below.

Following the diagnostic angiogram, a 0.035-inch Rosen curved guidewire (Cook) offered support to advance the guiding sheath up to the ostium of the renal artery to put a Penumbra 5Max aspiration catheter (Penumbra, Alameda, CA) in close contact with each embolus, which were successfully aspirated with complete restoration of renal arterial flow, as demonstrated in the final angiographic control (Figure 2b). An initial i.v. bolus of 5000 units of heparin was administered followed by continuous infusion of heparinized saline during the procedure through the guiding sheath. The patient was then continued on his usual oral anticoagulation scheme for atrial fibrillation.

## Outcome

Despite successful revascularization, the restoration of baseline renal function did not occur. Besides the fact that the 90 min limit of ischaemic tolerance of the kidney^1^ had already been exceeded, we believe that another major contributing factor to the poor clinical outcome was probably the previously reduced renal reserve, easily exacerbated by little additional damage to the kidney and precluding the expected compensatory hypertrophy by the non-affected tissue. ^1^ Nevertheless, the procedure did not cause any additional complications and the patient remained haemodynamically stable during the hospitalization.

## Discussion

ARI occurs mostly secondary to thromboembolism rather than *in situ* thrombosis, with the main risk factor being atrial fibrillation, but also includes myocardial infarction, infective endocarditis, aortic plaques and hypercoagulable status.^2,3^ The estimated incidence ranges between 0.007% ^4^ and 1.4%^5^, which is believed to be an underestimation owing to its unspecific presentation, leading to a misdiagnosis as either renal colic or pyelonephritis.^2^ Most patients complain of flank pain, nausea and vomiting, which might be associated with mild fever, acute elevation in blood pressure and, less frequently, oliguria.^2,3^ Regarding laboratory findings, leukocytosis, elevated serum creatinine and haematuria are frequent, while markedly elevated levels of serum and urinary lactate dehydrogenase are consistently present, the latter being particularly suggestive.^2,3^ A high grade of suspicion is required to make a prompt diagnosis and thus, in the appropriate clinical setting, a renal colour Doppler ultrasound should be performed after ruling out obstructive uropathy secondary to lithiasis by conventional ultrasound. It might accurately depict a global or segmental decrease in parenchymal flow that could help in assessing the permeability of the renal arteries and the vein, although its sensitivity is lower for segmental renal infarction.^5^ With a sensitivity of 80%, CECT is also an excellent modality,^2,3^ especially in cases with inconclusive Doppler evaluation. Conventional angiography provides a definite diagnosis.

There are no validated guidelines for the treatment of ARI, but it is consensual that reperfusion therapy should only be attempted if renal atrophy is absent.^2^ Antihypertensive and anticoagulation therapies are usually included, the latter being mandatory in atrial fibrillation.^2^ Surgery has a limited role in the non-trauma setting,^2,5^ although a bypass procedure might be considered in case of acute complete occlusion of the main renal artery.^6^ On the other hand, percutaneous endovascular therapies have been reported to be worthwhile, with some studies showing clear benefit with a delay of several days^2,7^ or even weeks.^8,9^ Nevertheless, poor outcomes with early intervention have also been reported,^1^ making the therapeutic window still unclear, especially because the literature is scarce. Moreover, regardless of successful reperfusion, renal function does not improve consistently.^2,5^ Endovascular revascularization procedures used so far with favourable outcomes include pharmacomechanical thrombectomy; selective intra-arterial thrombolysis alone or in combination with thrombus aspiration; and angioplasty, with or without stenting.^2,7,10–12^ So far, transcatheter thrombus aspiration has always been reported as a second-line technique combined with intra-arterial thrombolysis; ours is the first report that demonstrates its effectiveness alone.^10,11^

Unfortunately, regardless of complete revascularization, the outcomes in terms of restoration of renal function are inconsistent and seem to be even poorer for embolic occlusions than for thrombotic ones owing to unknown reasons.^13^ Nevertheless, until sufficient evidence to establish treatment guidelines for acute renal thromboembolism is available, we consider that endovascular revascularization should continue to be attempted at least in early diagnosed incomplete obstructions, especially in patients with a single functioning kidney. Percutaneous transcatheter thrombus aspiration alone might be effective and should therefore start being considered an alternative technique.

## Learning points

1. Prompt diagnosis and treatment are crucial for the outcome of ARI, as the precise therapeutic window remains unclear.
2. Renal Doppler ultrasound is the optimal initial diagnostic study, which, if inconclusive, should be followed by a CECT.
3. Despite the lack of validated guidelines, the addition of percutaneous endovascular procedures to medical therapy should be considered, at least in early diagnosed incomplete renal artery obstructions.
4. Among endovascular revascularization techniques, transcatheter thrombus aspiration alone might be an effective alternative.

## Consent

Informed consent could not be obtained despite exhaustive attempts. Nevertheless, the case has been sufficiently anonymized to protect patient identity.
